# Anthropogenic Drivers of Variation in Concentrations of Perfluoroalkyl Substances in Otters (*Lutra lutra*) from England and Wales

**DOI:** 10.1021/acs.est.1c05410

**Published:** 2022-01-11

**Authors:** Emily O’Rourke, Juliet Hynes, Sara Losada, Jonathan L. Barber, M. Glória Pereira, Eleanor F. Kean, Frank Hailer, Elizabeth A. Chadwick

**Affiliations:** † School of Biosciences, Cardiff University, Museum Avenue, Cardiff CF10 3AX, U.K.; ‡ Centre for Environment, Fisheries and Aquaculture Science (Cefas), Pakefield Road, Suffolk, Lowestoft NR33 0HT, U.K.; § U.K. Centre for Ecology and Hydrology, Lancaster Environment Centre, Library Avenue, Bailrigg, Lancaster LA1 4AP, U.K.

**Keywords:** per- and polyfluoroalkyl substances (PFASs), perfluoroalkyl carboxylic acids (PFCAs), perfluoroalkyl sulfonic acids (PFSAs), Eurasian otter (Lutra lutra), sentinel species, bioaccumulation, wastewater effluent, sewage sludge

## Abstract

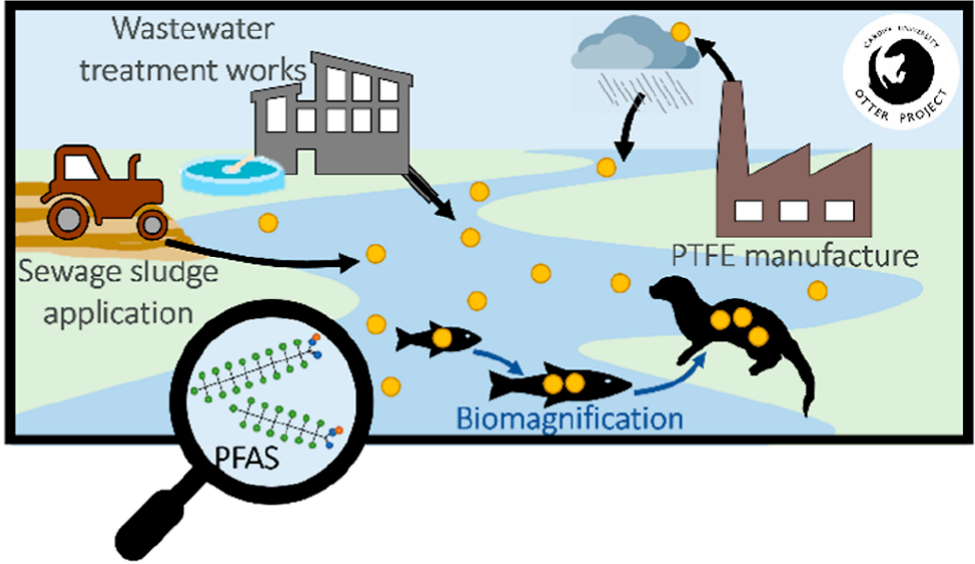

Per- and polyfluoroalkyl substances (PFASs) are ubiquitous environmental contaminants that have been linked to adverse health effects in wildlife and humans. Here, we report the presence of PFASs in Eurasian otters (*Lutra lutra*) in England and Wales and their association with anthropogenic sources. The following 15 compounds were analyzed: 10 perfluoroalkyl carboxylic acids (PFCAs), 4 perfluoroalkyl sulfonic acids (PFSAs), and perfluorooctane sulfonamide, in livers of 50 otters which died between 2007 and 2009. PFASs were detected in all otters analyzed, with 12/15 compounds detected in ≥80% of otters. Perfluorooctane sulfonate (PFOS) accounted for 75% of the ΣPFAS profile, with a maximum concentration of 6800 μg/kg wet weight (ww). Long-chain (≥C8) PFCAs accounted for 99.9% of the ΣPFCA profile, with perfluorodecanoic acid and perfluorononanoic acid having the highest maxima (369 μg/kg ww and 170 μg/kg ww, respectively). Perfluorooctanoic acid (PFOA) concentrations were negatively associated with the distance from a factory that used PFOA in polytetrafluoroethylene manufacture. Most PFAS concentrations in otters were positively associated with load entering wastewater treatment works (WWTW) and with arable land, suggesting that WWTW effluent and sewage sludge-amended soils are significant pathways of PFASs into freshwaters. Our results reveal the widespread pollution of British freshwaters with PFASs and demonstrate the utility of otters as effective sentinels for spatial variation in PFAS concentrations.

## Abstract 2

Perfluoroalkyl substances (PFASs) in otters suggest the widespread pollution of British freshwaters with wastewater, sewage sludge, and polytetrafluoroethylene (PTFE) manufacture.

## Introduction

**Figure 1:**
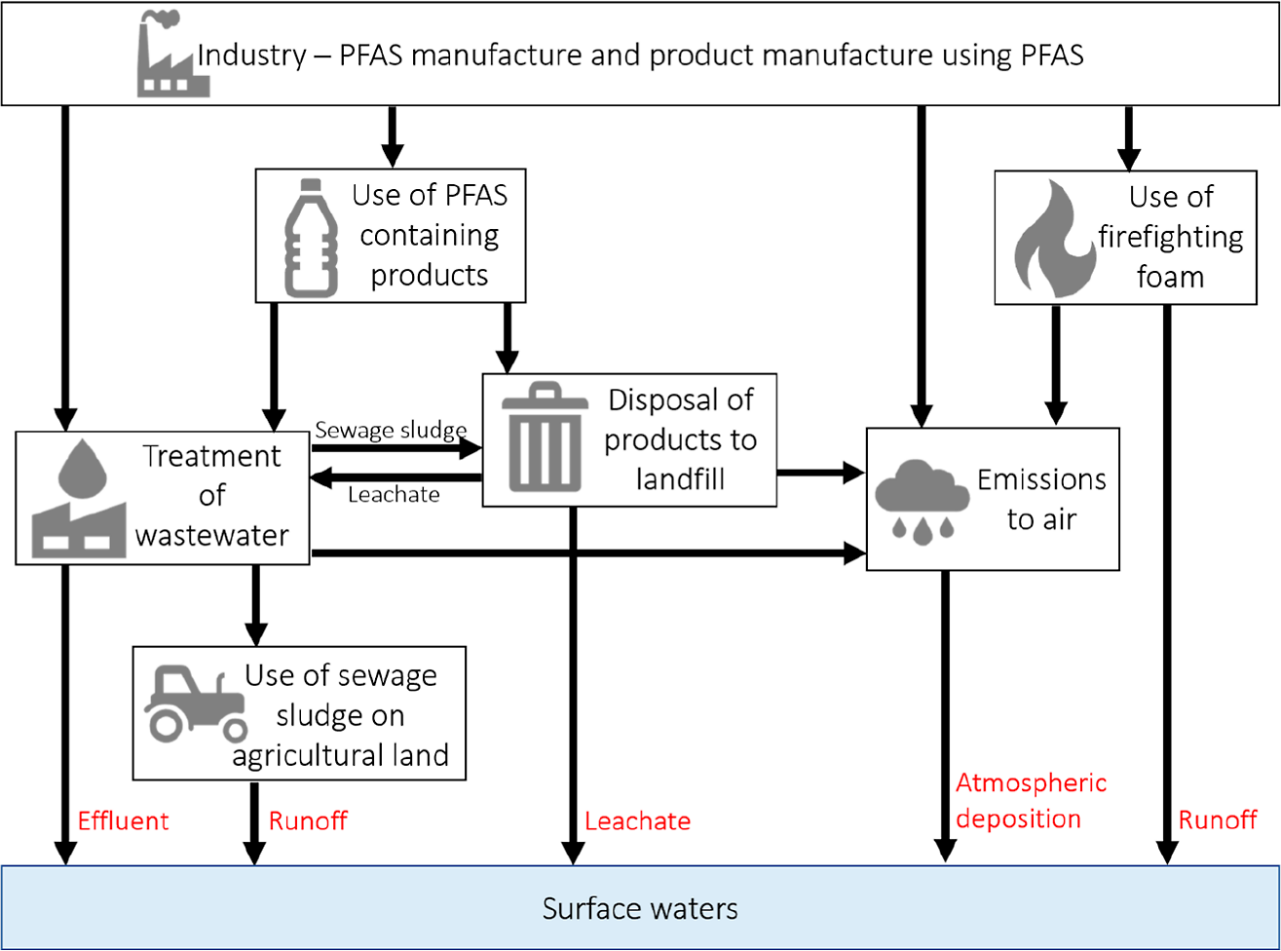
Sources of per- and polyfluoroalkyl substances (PFASs) in surface freshwaters.^7,11−15^

Per- and polyfluoroalkyl substances (PFASs) are a large family of highly fluorinated aliphatic anthropogenic chemicals, which have been used since the late 1940s in a wide variety of industrial and commercial applications.^1,2^ The use of PFASs has drawn increasing concern and regulatory interest due to accumulating evidence about their persistence in the environment, bioaccumulative potential, and toxicity in both wildlife and humans.^3−7^ The perfluoroalkyl moiety, common to all PFASs, imparts hydrophobic, oleophobic, and temperature-resistant properties to the compounds at enhanced levels compared to hydrocarbon analogues.^8^ These properties make PFASs desirable for use in surfactants and surface protectors. However, this moiety also results in very stable substances that resist chemical, thermal, and biological degradation and thus PFASs persist and accumulate in the environment.^7,9^ PFASs are highly soluble in water,^10^ and the major pathways into the environment are via landfill leachate,^11^ wastewater effluent from industry and domestic sources,^12^ run off from sewage sludge-amended soils,^13^ and run off after the use of PFAS-based firefighting foam^14^ (Figure 1). To a lesser degree, PFASs are emitted into air.^15^ The more volatile PFASs, such as fluorotelomer alcohols (FTOHs), are highly mobile in air and can be transported long distances in the atmosphere.^7^ Contamination of surface waters and marine systems is an inevitable consequence, and PFASs have been detected ubiquitously across the globe, even in remote locations such as the Arctic and mid-ocean islands.^16^

Perfluoroalkyl acids (PFAAs), a non-polymer perfluorinated sub-group of the PFAS family, are of particular concern. They have been produced and used extensively, resulting in thousands of tonnes of PFAAs being released into the environment.^17^ Additionally, PFAAs are the terminal degradation products of other PFASs, such as FTOHs, adding to the environmental burden. Globally, high concentrations of PFAAs have been recorded in the environment, resulting in the exposure of wildlife and humans to PFAAs through consumption of fish and drinking water.^18^ Perfluorooctane sulfonate (PFOS) and perfluorooctanoic acid (PFOA) are the most extensively analyzed PFAA. Toxicological studies have shown PFOS and PFOA to have negative impacts on the reproduction, liver function, metabolism, and immune system in both animals and humans.^19,20^ As a result of concerns for human health, since 2000, a series of voluntary industry initiatives (e.g., PFOA Stewardship agreement^21^) and legislation (e.g., Stockholm Convention^22^) have restricted the manufacture and use of PFOS and PFOA. Some studies have shown a decline in PFOS and PFOA concentrations as a result of these restrictions; however, this is not a universal finding,^23^ and concentrations of PFOS are regularly recorded above the Environmental Quality Standards (EQS; part of EU Priority Substances Directive 2013/39/EU^24^) for water and fish in England.^25−27^ Additionally, there is concern regarding the increasing concentrations of short-chain PFASs used as replacements for PFOS and PFOA.^28^ Top predators are at greater risk from these biomagnifying contaminants than other trophic levels. Eurasian otters (*Lutra lutra*) are non-migratory predators with a predominantly piscivorous diet^29^ and have been shown to be an effective sentinel for some contaminants.^30,31^ Their wide distribution across Europe, Asia, and northern Africa makes them a good candidate as a sentinel for contaminants across countries and continents.^32^ Within Britain, otters are the top predator of freshwater ecosystems and are, therefore, likely to be a good indicator of exposure to contamination via the manufacture, use, and disposal of PFAS-containing products.

For risk management and policy development, there is a need to understand the major pathways of PFASs into the British freshwater environment, and the impacts of legislation on both pathways and concentrations. In this study, we report concentrations for 14 PFAAs and one precursor PFAS (perfluorooctane sulfonamide) in 50 Eurasian otters (*Lutra lutra*) across England and Wales. We hypothesize that (1) PFASs will be widespread in otters across England and Wales and (2) measured concentrations of PFASs in otter tissues will reflect the spatial variation in anthropogenic sources including landfill, wastewater, agriculture, industry, and urban areas.

## Materials and Methods

### Target Compounds

Fifteen compounds were targeted in this study as follows: four perfluoroalkyl sulfonic acids (PFSAs) of 4, 6, 8, and 10 carbons in length, ten perfluoroalkyl carboxylic acids (PFCAs) increasing in a carbon chain length from 5 to 14, and perfluorooctane sulfonamide (PFOSA) (Table 1).

**Table 1:** List of Determinands: Details Include the Chemical Name, CAS-Number, Abbreviation, Carbon Number (*C*_n_), and Limit of Quantification (LOQ) Measured in μg/kg Wet Weight Achieved During This Study^a^

chemical name	CAS-number	abbreviation	*C*_n_	LOQ
perfluoroalkyl sulfonic acids (PFSAs)				
perfluorobutane sulfonic acid	375-73-5	PFBS	4	0.05
perfluorohexane sulfonic acid	355-46-4	PFHxS	6	0.05
perfluorooctane sulfonic acid	1763-23-1	PFOS	8	0.05
perfluorodecane sulfonic acid	335-77-3	PFDS	10	0.05
perfluoroalkyl carboxylic acids (PFCAs)				
perfluoropentanoic acid	2706-90-3	PFPeA	5	0.05
perfluorohexanoic acid	307-24-4	PFHxA	6	0.05
perfluoroheptanoic acid	375-85-9	PFHpA	7	0.05
perfluorooctanoic acid	335-67-1	PFOA	8	0.05
perfluorononanoic acid	375-95-1	PFNA	9	0.05
perfluorodecanoic acid	335-76-2	PFDA	10	0.05
perfluoroundecanoic acid	2058-94-8	PFUnA	11	0.05
perfluorododecanoic acid	307-55-1	PFDoDA	12	0.1
perfluorotridecanoic acid	72629-94-8	PFTrDA	13	0.1
perfluorotetradecanoic acid	376-06-7	PFTeDA	14	0.1
precursor compound perfluorooctane sulfonamide (PFOSA)				
perfluorooctane sulfonamide	754-91-6	PFOSA	8	0.05

a Nomenclature follows that of Buck et al.^1^

### Otter Samples and Associated Biotic Data

Otters found dead (largely road traffic casualties) were collected as part of the Cardiff University Otter Project and stored frozen at −20 °C prior to post-mortem examination. For each individual, the location (National Grid Reference) and date found were recorded by the finder, and a range of biometric data (including sex, age-class, length, weight, and reproductive status) were recorded during a standardized post-mortem examination (www.cardiff.ac.uk/otter-project). A body condition score was calculated from the length and weight using the Peig and Green^33^ scaled mass index (SMI). Tissue samples, including liver, were collected, wrapped in aluminum foil, and archived in individual grip seal bags at −20 °C.

In order to focus on spatial variation, we restricted biotic and temporal variation. We excluded juvenile and sub-adult otters (based on body length and reproductive features; excluding males <3 kg and females <2.1 kg, as well as any males with baculum length <60 mm, and females with no evidence of reproduction, that is, immature uterus, no placental scarring and teats not prominent). We additionally excluded otters with gross evidence of decay based on textural changes to the tissues, discoloration, smell, visible bacterial invasion, or fly eggs/larvae; therefore, only retaining otters deemed to be freshly dead. We further limited sample selection to only include otters found between 2007 and 2009, in order to restrict potential change over time. During this time period PFOS and PFOA (the two most widely used PFAS) were being phased out and replacements were emerging; this time period is therefore a likely turning point in time trends (which were not the focus of this study, but results can be used as a baseline for future studies). Samples from 282 fresh adult otters, which died between 2007 and 09, were available; further selection was made on the basis of spatial data (see below).

### Spatial Data Sources and Extraction

We collated data describing point and diffuse anthropogenic sources of PFASs in the freshwater environment (Table 2). All spatial data and the location of death of all 282 potential otters were mapped as shapefiles in ArcMap GIS (10.2.2). The otter’s home range along water courses varies between 5 and 40 km,^34,35^ and otters are also known to travel over land between water courses, thus the potential area of exposure to pollutants might extend some distance from the known location of death. Therefore, each otter location was used as the center point for a circular area, 10 km in radius, to create polygons representing the likely range over which each otter might have been exposed. The mean value for PFOS discharge, wastewater treatment works (WWTW) load, and rainfall, for each 10 km radius area, were extracted using isectpolypoly and isectpolyrst tools from the Geospatial Modeling Environment (version 0.7.2). Percentage coverage of arable land, pastoral land, urban area, and landfill site area in each 10 km radius circular area were calculated using the ArcMap tabulate intersection tool. The linear distance from the location of death for each otter to the identified point source of PFOA [AGC Chemicals Europe, Ltd., located on the northwest coast of England, which used PFOA in PTFE manufacture at the time these otters were sampled (2007–09): 53°52′59″N, 003°00′03″W] was measured using an ArcMap join tool. Samples were then ranked by each spatial data set, and labeled using quantiles, to enable stratified sample selection that included otters from across the data distribution for each variable. Fifty otters were selected for analysis, with a balanced sex ratio (*n* = 23 female, *n* = 27 male). The spatial distribution of selected individuals is mapped in Figure S1 and the range of values for biotic and spatial variables used in statistical modeling is shown in Figure S2. It should be noted that some otters were found in coastal locations and may have been exposed to PFASs from marine as well as freshwater systems. Other (unpublished) research from our group suggests, however, that marine prey represents only a small proportion of diet even among coastal otters in England and Wales,^36^ and whether coastal or inland, the location of the otter is still representative of the exposure in the local environment.

**Table 2:** Biotic (^B^) and Spatial (^S^) Variables Pertinent to Testing the Association of PFAS Concentrations in Otters to Anthropogenic Sources

variable	detail	data source	GLMs variable included in
^B^ otter length	total length, nose to tip of tail (mm). Included to control for differences in concentration as a result of body size. Correlated with sex due to sexual dimorphism	measured at post-mortem examination by Cardiff University Otter Project (CUOP)	all
^B^ otter sex	male (*n* = 27) or female (*n* = 23). Differences between male and female may include offloading of pollutants to offspring for females	determined at post-mortem examination by CUOP	none, due to collinearity with length
^B^ otter body condition	scaled mass index (SMI) estimate of body condition (using Peig and Green^34^). Test for the potential impact of PFASs on body condition or vice versa	calculated from length and weight measurements taken during post-mortem examination by CUOP	all
^S^ percentage landfill area	percentage of land in 10 km buffer of otter that has historic or currently used landfill sites. Source of PFASs via the disposal of consumer products	Natural Resources Wales^47,48^ and Environment Agency^49,50^	all
^S^ percentage urban area	percentage of urban and suburban land in the 10 km radius of otter. Source of PFASs via the use of consumer products, and the use of PFAS-based firefighting foam, including at airports	U.K. Centre for Ecology and Hydrology—Land Cover Map 2007^51^	none, due to collinearity with proportion landfill area
^S^ mean wastewater treatment works load	mean load entering WWTW, measured in population equivalent (PE), within a 10 km radius around each otter. Source of PFASs via the disposal of industrial and domestic wastewater	European Environment Agency^52^	all
^S^ percentage pastoral land	percentage of improved grassland within a 10 km radius around each otter. Potential proxy for the application of sewage sludge on pastoral land	U.K. Centre for Ecology and Hydrology—Land Cover Map 2007^51^	all
^S^ percentage arable land	percentage of arable land within the 10 km radius around each otter. Potential proxy for the application of sewage sludge on arable land	U.K. Centre for Ecology and Hydrology—Land Cover Map 2007^51^	all
^S^ rainfall	mean rainfall (mm) within the 10 km radius around each otter from years 2006–2009. Source of PFASs via atmospheric deposition or dilution within river	Met Office^53^	none, due to collinearity with proportion arable land
^S^ distance to factory producing PTFE	linear distance from each otter to a factory using PFOA at a time of sampling (2007–09) in PTFE manufacture (AGC Chemicals Europe Ltd, Lancashire)	calculated using ArcGIS join tool to factory location^54^	PFOA only
^S^ PFOS discharge to water	binary variable, discharge reported, or no discharge reported within a 10 km radius around each otter	pollution inventories for Wales and England^55,56^	PFOS only

### Analytical Determination

Frozen liver subsamples were sent to Centre for Environment, Fisheries, and Aquaculture Science (Cefas), Lowestoft, UK, for analysis, according to the method of Verreault et al.^37^ Before extraction, samples were thawed and homogenized. 1 g of samples were spiked with 20 μL of a mixture of isotopically mass-labeled recovery/internal standards (ISTDs) in methanol containing 0.2 ng/μL of each ISTD (^13^C_2_-PFHxA, ^13^C_4_-PFOA, ^13^C_5_-PFNA, ^13^C_2_-PFDA, ^13^C_2_-PFUnDA, ^13^C_2_-PFDoDA, ^13^C_2_-PFTeDA, ^13^C_8_-PFOSA, ^18^O_2_-PFHxS, and ^13^C_4_-PFOS, all from Wellington, Guelph, Canada) in polypropylene tubes. The samples were extracted twice with 5 mL of acetonitrile in an ultrasonic bath (15 min, room temperature). Concentrated extracts underwent dispersive clean-up on 25 mg of graphitized carbon (Supelclean ENVI-Carb 120/400, Supelco, Sigma-Aldrich, Stockholm, Sweden) and 50 μL of glacial acetic acid in Eppendorf tubes. Aliquots of 0.5 mL of the cleaned-up extracts were diluted with 0.5 mL of 4 mM aqueous ammonium acetate and kept at 4 °C until the day of analysis. The extracts were allowed to warm to room temperature, vortex mixed, and centrifuged before the clear solution was transferred to an autoinjector vial, together with 10 μL of a mixture of isotopically mass-labeled injection standards containing 500 ng/μL of ^13^C_8_-PFOA and ^13^C_8_-PFOS. The analysis of PFASs was done by isotope dilution and performed using an ultra-performance liquid chromatograph Acquity (Waters Ltd, Elstree, Hertfordshire, UK) using a BEH C18 analytical column (50 mm × 2.1 mm and 3.5 μm particle size) from Waters. A column XBridge C18 (column 50 mm × 2.1 mm and 1.7 μm particle size) from Waters was used as an isolator column. The UPLC system was coupled to a TQ MS Xevo triple quadrupole mass spectrometer (Waters Ltd, Elstree, Hertfordshire, UK), using an electrospray ionization (ESI) probe in the negative mode. When isomers were present in samples, only the linear isomer was quantified against the linear PFASs present in standards, and results are reported for the linear isomer only, as recommended by Berger et al.^38^ For quality assurance purposes, a blank and reference material sample (flounder tissue from sixth Interlaboratory Study on PFASs in Environmental Samples 2013) were analyzed with every 10 samples. Limits of quantification for each of the fifteen determinands are shown in Table 1.

### Data Analysis

For the purposes of statistical analysis, samples below the limit of quantification (LOQ) were assigned 0.5 × LOQ. All statistical analyses were carried out in *R* (version 4.0.3).^39^ To explore the biotic and abiotic drivers of the contaminant load using a multivariate approach, generalized linear models were fitted, with concentrations of each contaminant as the dependent term, and biotic and spatial variables as independent terms (Table 2). Perfluoropentanoic acid (PFPeA), perfluorohexanoic acid (PFHxA), and perfluoropentanoic acid (PFHpA) were excluded from these analyses as detection frequencies were too low (0, 12, and 42%, respectively) to provide adequate data for modeling purposes.

Initial exploration of data distributions of the dependent variables showed that measured concentrations were typically highly skewed, with the exception of PFNA (see Figure S2). Therefore, preliminary models fitted using untransformed concentration data with a Gaussian error family and identity link function were compared with identical models using a Gaussian error family and log link, Gamma error family and log link, and log-transformed concentration data with the Gaussian error family and identity link. Model residuals were compared to evaluate normality, homoscedasticity, and leverage, and resulted in the selection of log-transformed data with a Gaussian error family and identity link function for all models, except PFNA, for which raw data with a Gaussian error family and log link function was optimal. Variance inflation factors were calculated for all covariates in the starting models, using the corvif function in the Car package,^40^ and consequently sex, percentage of urban land, and rainfall were removed from all starting models (see Table 2). All models included otter length, otter body condition, the log of percentage landfill (log values were used to improve model fit), mean wastewater treatment loading, percentage of pastoral land, and percentage of arable land (within the 10 km radius area around each otter). Additionally, in the model for PFOA, distance to the PTFE factory was included, and in the model for PFOS, PFOS discharge (as a binomial variable: discharge reported, or no discharge reported) was included. PFOS discharge as a continuous variable (mean kg/year released in the 10 km radius of otter) was not included in the starting model following preliminary model checks because zero inflation caused model assumptions to be violated.

Determination of the most important variables was achieved using multimodel inference: independent variables were standardized using the standardize function in the Arm package,^41^ the dredge function in the MuMIn package^42^ was then used to rank models by AICc, and model averaging was applied to models where delta AICc was <2.^43^ The full average method, whereby parameter averages are calculated using the total number of top models and setting the parameter to zero in models it does not appear in, was used to determine model estimates, as it is deemed more appropriate when the study aim is to determine which independent variables have the strongest effect on the dependent variable.^44^ The most important associations were determined as those which either appeared in all top models (relative importance [RI] = 1) regardless of probability, or where RI was >0.5 and the relationship was statistically significant (*p* < 0.05).^45^ For each compound, the average model was used to derive model predictions (using the “predict” function in *R*), while controlling for other retained variables to their mean value (length = 1095 mm, condition = 6.022, percentage landfill (logged) = 0.46%, average WWTW load = 15731 PE, percentage arable land = 34.34%, and percentage pastoral land = 27.83%).

The association of PFOA with the factory producing PTFE was further tested using a Mann–Whitney test of the difference between concentrations in otters north of the PTFE manufacturing facility (prevailing winds typically from west and southwest^46^), versus those south of the factory. A non-parametric analysis was used because the assumption of normally distributed data for the parametric alternative (two sample *t*-test) was violated.

## Results and Discussion

**Figure 2:**
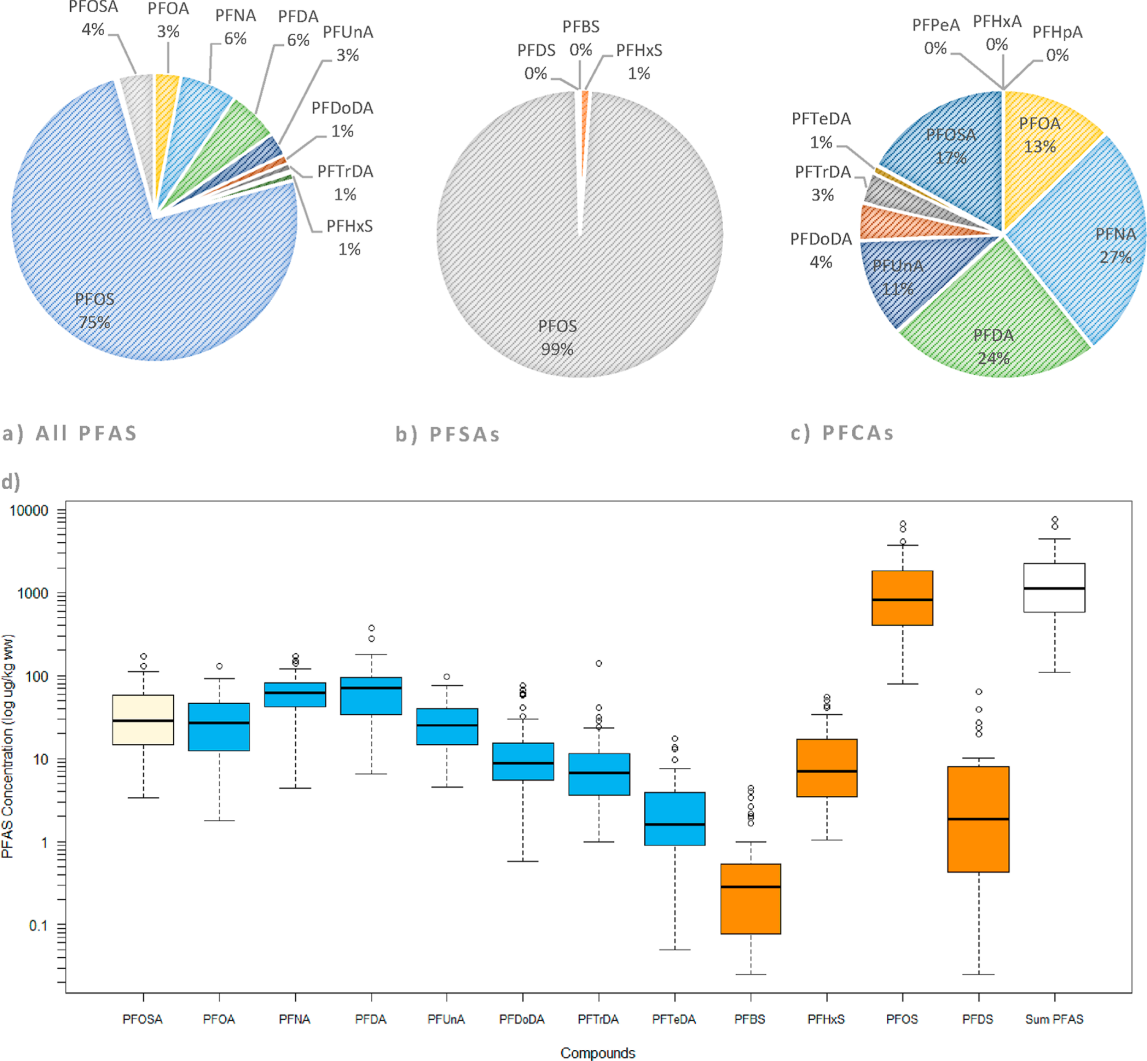
Proportion of individual substances in relation to the total of (a) all PFASs (PFBS, PFDS, PFPeA, PFHxA, and PFHpA represented 0% of the profile and are consequently not shown) (b) perfluoroalkyl sulfonic acids (PFSAs), and (c) perfluoroalkyl carboxylic acids (PFCAs). Compounds are denoted by their abbreviation see Table 1 for full names. (d) Concentrations of compounds with detection frequency 80% and above. Compounds are denoted by their abbreviation. Concentrations are recorded in μg/kg ww and plotted on a log scale. Compounds are color coded; beige = perfluorooctane sulfonamide (PFOSA), blue = perfluoroalkyl carboxylic acids (PFCAs), orange = perfluoroalkyl sulfonic acids (PFSAs), and white: sum PFASs; and presented in order of the carbon chain length within each group. Concentrations are presented as a boxplot; the thick black line indicates the median concentration, the lower and upper extents of the box indicate the 25th (*Q*1) and 75th (*Q*3) percentiles of the data distribution, whiskers show the lowest and highest values excluding outliers, and circles indicate outliers (1.5× the interquartile range).

This is the first report of PFASs in Eurasian otters from Britain: detectable concentrations of PFASs were found in all livers analyzed. ΣPFAS concentrations ranged from 109 μg/kg wet weight (ww) to 7652 μg/kg ww, with PFOS accounting for the highest proportion of this profile (75%, Figure 2a). Our models show that a spatial variation of PFASs in otters is associated with anthropogenic sources; here, we first explore the concentrations detected in otters and then discuss the significant associations with sources.

### PFAS Concentrations and Possible Health Impacts

Twelve of the fifteen compounds analyzed were detected in ≥80% of samples (all PFSAs, 7 PFCAs and PFOSA). Of the remaining 3 PFCAs, PFHpA (C7), and PFHxA (C6) were detected in 42 and 12% of the samples, respectively; while PFPeA (C5) was not detected at all (Table S1).

#### Perfluoroalkyl Sulfonic Acids

PFOS dominated the ΣPFAS profile (Figure 2a), numerous other studies have also found PFOS to be the predominant PFAS analyzed,^26,57−62^ reflecting the widespread and extensive use of PFOS in consumer products, pesticides, and aqueous film-forming firefighting foam (AFFF), and its high bioaccumulative potential. In this study, PFOS concentrations ranged from 78.8 to 6,800 μg/kg ww, which is comparable to the concentrations seen in Eurasian otters from freshwater systems in Sweden collected between 2005 and 2011 (32–7350 μg/kg ww),^60^ and higher than concentrations seen in otters feeding primarily in marine systems [Eurasian otters in Norway (2010), 63–370 μg/kg ww,^60^ and sea otters (*Enhydra lutris*) from the Californian coast (1992–2002), <1–884 μg/kg ww.^58^] A European study on PFAS concentrations in apex predators found buzzards (*Buteo buteo*), which typically feed on terrestrial prey, to be the least contaminated when compared to Eurasian otters and marine apex mammals [harbor seals (*Phoca vitulina*), gray seals (*Halichoerus grypus*), and harbor porpoises (*Phocoena phocoena*)] from the same countries.^32^ Differences in the top predator accumulation of PFOS between freshwater, marine, and terrestrial systems are likely to reflect a complex suite of factors, including proximity to sources, differing food webs, and species specific differences in bioaccumulation and metabolism.^5^ Recent research suggests that freshwater predators may have some of the highest concentrations^32^ and although terrestrial species living in close proximity to sources can show very high concentrations,^63^ PFASs are highly soluble and therefore the predominant exposure pathway into the environment is via water.^17^ Concentrations in freshwater species may be high relative to those in marine systems due to their closer proximity to a range of sources, including effluent, leachate, and runoff.^16^

Among the other PFSAs, the high detection of perfluorohexane sulfonic acid (PFHxS) and perfluorodecane sulfonic acid (PFDS) was expected due to a greater bioaccumulation of long-chain PFSAs (C6+), compared to short-chain compounds.^1^ The high detection frequency of perfluorobutane sulfonic acid (PFBS, 80%), a short-chain compound (C4), reflects the increase in its use as a replacement for PFOS since 2000. Short-chain compounds, such as PFBS, were considered safer alternatives to long-chain compounds because of their presumed lower bioaccumulative potential and toxicity.^64^ While studies on fish and invertebrates have failed to detect PFBS,^26,65^ detection in mammalian top predators has been reported.^60,61,66^ The relatively low concentrations of PFBS found in the current study are likely to reflect both the lower bioaccumulative potential of short, compared to long chain, PFAAs, as well as the more recent introduction of PFBS. Increases in concentration over time have been found in marine mammals between 2002 and 2014,^66^ and it seems likely that an increased usage of PFBS since our sampling period (2007–09) will have led to an increase in the PFBS pollution in Britain. Evidence is growing that short-chain compounds have toxicological effects similar to those resulting from long-chain PFASs,^7^ and continued monitoring is therefore important. Given the low detectability in fish, monitoring using a top predator, such as the otter, is likely to make a valuable contribution to understanding exposure and risk in freshwater systems. Due to their high trophic level, otters are excellent sentinels for chemicals that bioaccumulate and biomagnify in the environment. For substances which do not bioaccumulate or biomagnify (or do so to a lesser degree, such as the short-chain PFAAs) detection presents an additional challenge. Species such as the otter are typically longer lived and range over larger areas than non-migratory freshwater fish, and thus integrate chemicals over space and time—providing an effective mechanism for quantifying contamination with substances that may be non-detectable in fish, such as PFBS.

#### Perfluoroalkyl Carboxylic Acids

ΣPFCA concentrations ranged between 24.8 and 764 μg/kg ww, with the highest concentrations seen in otters from East Anglia, a geographical area in the southeast of England. Concentrations of the long-chain, C8-14, compounds accounted for 99.9% of the ΣPFCA profile, with PFDA and PFNA accounting for 61% of that (Figure 2b). A higher detection of long-chain compounds (C8+) was expected due to greater commercial use, and C8+ PFCAs being more bioaccumulative than short-chain compounds.^1^ Across the group, median concentrations increased with the chain length to a peak at PFDA (C10) and then declined (Figure 2d); a predominance of odd over even long-chain PFCAs (typically seen in marine biota^57,67^) was not observed and is consistent with findings from Eurasian otters in Sweden.^60^ The predominance of the odd chain length PFCAs has been attributed to the degradation of FTOHs. FTOHs breakdown to form equal quantities of two adjacent odd and even chain PFCAs, for example, 10:2 FTOH degrades to PFDA (C10) and PFUnA (C11) in equal amounts but because the longer chain PFCA (PFUnA in this example) is more bioaccumulative, it predominates over the shorter, even chain length PFCA in biota.^16^ FTOHs can be transported long distances in the atmosphere and therefore likely contribute greatly to the elevated proportions of odd chain PFCAs in marine biota, whereas in freshwater biota, living closer to the direct sources of PFCAs, this pattern is obscured.^16^

The median concentrations of PFDA (C10) and PFNA (C9) were more than twice that of PFOA (70.9, 63.1, and 27.2 μg/kg ww, respectively). Globally, PFOA (C8) was used and emitted in the greatest quantities, and abiotic sampling reflects this with higher PFOA concentrations observed;^17^ however, PFNA is often more prevalent in biota studies using the liver tissue.^57,59^ This is due to differing hepatic kinetics between ≤C8 and ≥C9 PFCAs; the aqueous solubility of ≤C8 allows for urinary excretion, whereas the relative hydrophobicity of C9–C11 PFCAs favors biliary enterohepatic recirculation and therefore storage in the liver.^68^ The difference in predominant compounds between abiotic and biotic samples demonstrates that production quantity does not necessarily correlate with concentrations detected in biota. This emphasizes the importance of biomonitoring and including toxicokinetics in risk assessment, rather than solely examining emissions, to understand the bioavailability and bioaccumulation of compounds, and consequently the potential risk to wildlife.

#### Perfluorooctane Sulfonamide

PFOSA was the only non-terminal compound included in the study (it degrades to form PFOS in the environment and in wildlife^69^). PFOSA was detected in all samples and its median concentration (28.8 μg/kg ww) ranks it fourth highest among the determinands tested (Figure 2d). PFOSA has been found in high concentrations in a number of wildlife studies. In cetaceans and fish studies, PFOSA concentrations in the liver are often similar or even higher than PFOS concentrations,^57,69^ whereas in Carnivora species PFOS concentrations tend to be many times higher than PFOSA concentrations (as was the case in the current study on otters). Evidence suggests there is a phylogenetic difference in the ability to metabolize PFOSA to PFOS, with rapid biotransformation in Carnivora.^69^ High concentrations of PFOSA in fish, and the subsequent biotransformation into PFOS in otters potentially represents an important route of exposure to PFOS for otters.

#### Potential Health Impacts

A detailed evaluation of health effects was beyond the scope of this study, and as no toxic thresholds have been determined for any PFAS in Eurasian otters, it is difficult to directly evaluate the potential relevance of the concentrations seen here in relation to Eurasian otter health. However, studies on other wild mammals consistently show PFAS concentrations negatively impacting biomarkers of exposure and effect.^70^ We retrospectively screened post-mortem records of all 50 otters for any abnormalities. We identified three otters with enlarged adrenal glands, which can be a sign of disease, two of these otters had very high ΣPFAS concentrations (the highest, and fifth highest). We also identified one male otter with unilateral cryptorchidism, which has been linked to environmental pollution;^71^ this otter had the fifth highest ΣPFAS concentration of the male otters (seventh overall). Laboratory studies have suggested associations between PFAA exposure and immunotoxicity in animals.^72^ While it is challenging to be certain of a link between the PFAS exposure and immune system effects in field studies due to the large number of confounding variables, which may impact immune system health, some studies have shown an association between immune system health and environmentally relevant concentrations of PFAA. For example, in sea otters, a study found PFOA and PFOS concentrations to be significantly higher in otters that died of infectious disease than non-diseased animals.^58^ Seven otters in our study exceeded the median PFOA concentration seen in the diseased sea otter group (68 μg/kg ww), and all otters exceed the median PFOS concentration of the diseased group (41 μg/kg ww). While sea otters and Eurasian otters may have different sensitivities to the health impacts of PFASs, and our otters did not show signs of disease, apart from those few mentioned above, the comparably high concentrations seen in our study are cause for concern. Using a relative potency factor methodology, Bil et al.^73^ found that PFCAs and PFSAs with 7 to 12 perfluorinated carbons are equally or more potent than PFOA for liver endpoints. In this study, concentrations of PFOS (C8), PFNA (C9), and PFDA (C10) were all higher than that of PFOA. Consequently, it is possible that PFASs are adversely impacting otter health, especially when the combined effect of exposure to multiple PFASs is considered.

Health effects have been shown across multiple PFASs, including short-chain PFAAs, such as PFBS and PFHxA, which were considered safer alternatives to PFOS and PFOA. However, of all the PFASs, only a small fraction have been tested for their harmful effects.^7^ The ubiquitous presence of PFASs in the otters tested in our study, and therefore in the British environment, supports the need for a class-based approach to regulating PFASs. Proceeding with testing and legislation substance-by-substance has the potential to take too long and have detrimental impacts on wildlife and human health.^7^

### Anthropogenic Drivers of Variation in Contaminant Concentrations

General linear models were used to explore the associations between PFAS concentrations and anthropogenic sources. Full model outputs are provided in Table S2. 10 of the 12 PFASs modeled showed a significant association with at least one source; significant sources are discussed here: WWTW load, percentage arable land (in 10 km radius of each otter), and, in the case of PFOA, distance from the PTFE manufacturing factory. Percentage landfill area and percentage pastoral land were retained in some models, but were not significant or important (RI = 1) in any models. PFOS discharge within a 10 km radius of the otter was not significant or important in the PFOS model.

#### PTFE Manufacturing Facility

**Figure 3:**
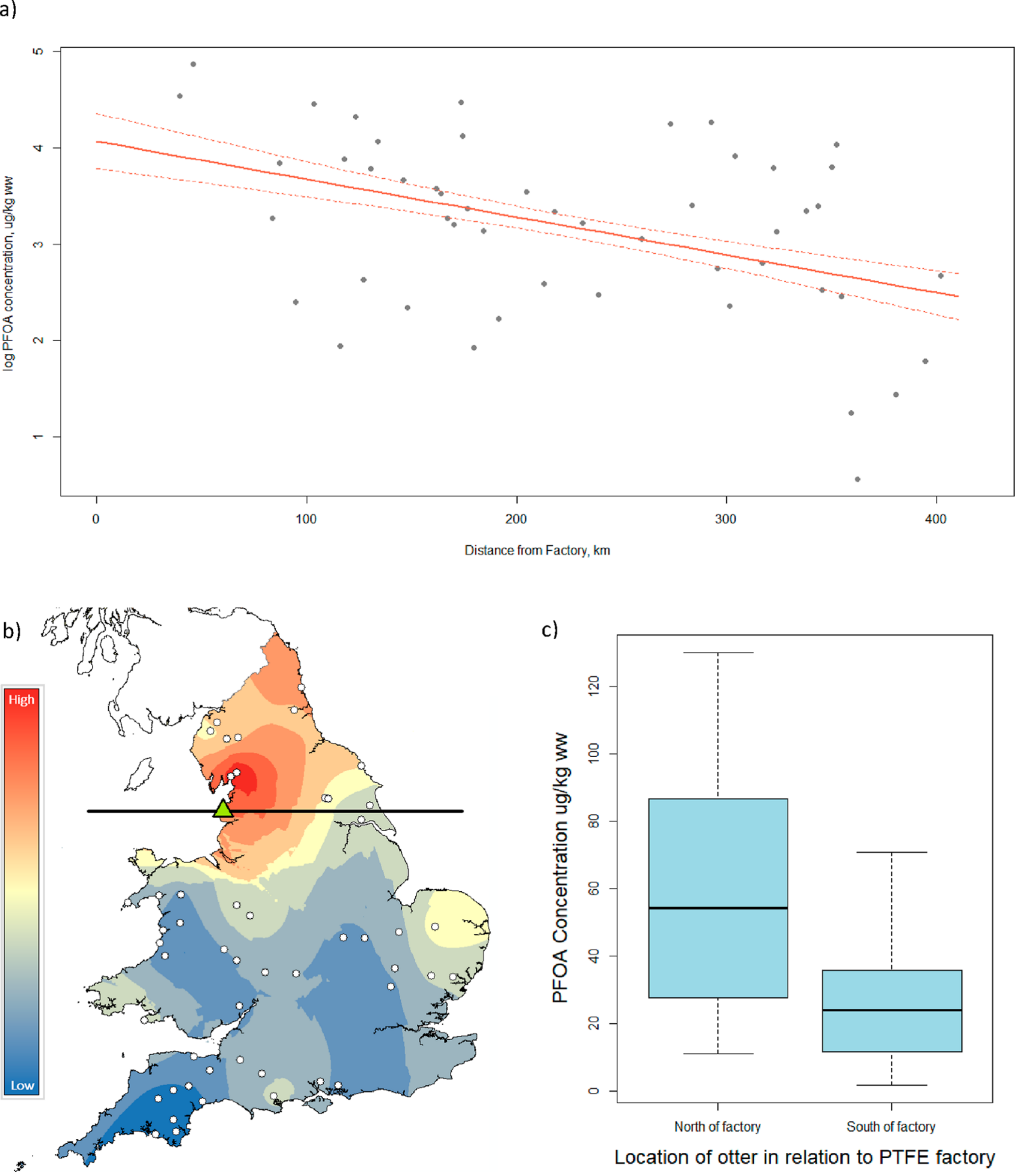
(a) Model-predicted PFOA concentrations (red lines, ± SE) with distance from the factory producing PTFE. Other variables in the model are controlled (WWTW and arable land, see statistical methods). (b) Heatmap showing PFOA concentrations, measured in μg/kg ww. Red indicates high values and dark blue indicates low. White dots show locations of otters used in analysis. Green triangle shows the location of the factory, black line indicates the latitude of the factory. (c) Concentrations of PFOA in otters north and south of factory latitude line. Concentrations are measured in μg/kg ww. Concentrations are presented as a boxplot; the thick black line indicates the median concentration, the lower and upper extent of the box indicate the 25th (*Q*1) and 75th (*Q*3) percentiles of the data distribution, whiskers show the lowest and highest values excluding outliers.

Historically, the single largest use of PFCAs was as processing aids in the manufacture of fluoropolymers, with polytetrafluoroethylene (PTFE), produced using PFOA, accounting for the majority of world’s fluoropolymer consumption.^17^ One factory in England, AGC Chemicals Europe, Ltd., located on the Fylde Coast in Lancashire, used PFOA in PTFE manufacture at the time these otters were sampled (2007–09). PFOA showed a significant negative association with the distance from this factory, with the highest values (130 μg/kg ww and 93.1 μg/kg ww) seen within 47 km of the putative source, and the lowest values (1.76 μg/kg ww and 3.48 μg/kg ww) seen over 359 km away in the south of England (Figure 3a, averaged model: *z* = 2.701, *p* = <0.01). This supports previous evidence that PFOA is elevated near fluoropolymer-manufacturing plants.^74,75^ Visualizing this association indicates that the highest PFOA concentrations are seen in otters found north and east of the factory, which follows the direction of prevailing winds from the factory (Figure 3b,c). This difference between concentrations in otters north of the source, and those south, is statistically significant (Mann Whitney test, *W* = 354.5, *p* = <0.01). This result supports evidence that air dispersal with prevailing wind direction is an important pathway for PFOA contamination of the environment.^76^ PFAA are less volatile than other PFASs and therefore emissions to air form a much lower proportion of total PFAA pollution than discharge to water.^17^ However, in air samples collected from UK, Ireland and Norway, PFOA was ubiquitous in the particulate phase. Concentrations were highest at a semi-rural site in England, which was 20 km downwind of the same PTFE manufacturing facility (AGC Chemicals Europe, Ltd.), suggesting this factory was an important source for air concentrations detected.^77^ The factory phased out PFOA use in 2012 and started using C6 technology;^78^ future research should analyze whether the association of PFOA with this factory still persists, and whether spatial associations are now present with the replacement C6 compounds.

#### Load Entering Wastewater Treatment Works

Mean load entering WWTW (measured in population equivalent, PE) was a significant term in 8 of the 12 PFASs modeled (PFOA, PFDoDA, PFTeA, PFBS, PFHxS, PFOS, PFDS, and PFOSA). All had a positive correlation, with averaged coefficients suggesting an increase in the contaminant concentration of between 2.0 and 4.9% (SE between ±0.7 and 1.2%) for every 1000 PE increase in the WWTW load (Figure S3), supporting other studies, which have found that wastewater effluents are a significant exposure route for PFASs.^4,25^ Water containing PFASs enters WWTW from domestic sources, industrial sites, and landfill sites, and the PFASs are not effectively removed by conventional wastewater treatment processes.^12^ Moreover, PFAA concentrations in effluent have been shown to exceed those in influent due to the degradation of precursor PFAS compounds to terminal PFSAs and PFCAs during wastewater processing.^12,79^ Landfill (percentage of land used for landfill within the 10 km radius of each otter) was not significant in any models. PFAS concentrations in landfill leachate can be higher than in wastewater; however, it is likely that due to the large volume of water processed, WWTW release a greater mass of PFASs into the environment.^14^ The absence of any association also suggests that landfill leachate collection systems, and subsequent delivery to WWTW, are effective at reducing PFASs leaching from landfill sites in England and Wales. Landfill was highly positively correlated with the urban area (percentage of the urban land within the 10 km radius of each otter), therefore associations with urban area could not be explicitly tested. However, the lack of any significant association with landfill suggests that a direct runoff from urban areas (e.g., of PFAS-based firefighting foam and contaminated water) is likely not a significant pathway for PFASs into rivers.

#### Arable Land

Arable land (percentage of arable land within a 10 km radius of each otter) was retained in all averaged models and was a significant term in 4 of 7 PFCA models (PFDA, PFDoDA, PFTrDA, and PFTeDA) and all the PFSA models (PFBS, PFHxS, PFOS, and PFDS). In all cases, there was an increase in concentration with an increase in arable land, with averaged coefficients suggesting an increase in the contaminant concentration of between 1.2 and 4.0% (SE between ±0.4 and 0.8%) for every one percent increase in arable land in a 10 km radius around the otter (Figure S4). No association was found with pastoral land. The strong positive correlation for most PFASs analyzed may reflect sewage sludge application on crop land. Sewage sludge is formed during the treatment of wastewater and is recognized to be a major sink of PFAAs,^80^ with long-chain PFSAs and PFCAs having the highest sorption into sewage sludge.^13^ Consequently, run off after application is a known exposure route for local waterways.^13^ In the UK, approximately 75% of sewage sludge produced annually is applied to agricultural land, with most applied to arable crop land.^81^ Users of sewage sludge must abide by the sludge (use in agriculture) regulations, 1989, which stipulate that concentrations of heavy metals are measured in the sludge and receiving soil to ensure they are within permissible concentrations. There are currently no statutory limits for PFASs, although the Chemical Investigations Programme 3 (CIP3) is currently testing sludge for PFASs.^27^

It should be noted that arable land in Britain predominates in areas of low rainfall (we found a negative correlation between arable land and rain). Due to collinearity between variables, rainfall could not be included in our models, and we cannot rule out an association with rainfall rather than (or as well as) arable land. If atmospheric deposition was the predominant or only source of PFASs, we would expect to see a positive association with rainfall. Instead, we see a positive association with arable land and, by inference, a negative association with rainfall. Partitioning of PFASs from water to sediment is lower at sites with higher rainfall, due to the flushing of the contaminants downstream.^82^ In arable areas with low rainfall, therefore, increased PFAS inputs from sewage sludge, together with limited flushing, may jointly be driving the higher PFAS concentrations detected. Further, more spatially explicit, research is needed to fully disentangle these potential drivers.

Overall, our data show that PFASs are ubiquitous in otters and the freshwater ecosystems in England and Wales, and concentrations in otters are reflective of anthropogenic sources. The negative correlation of the PFOA concentration with distance from the PTFE manufacturing facility shows that the use of PFOA in product manufacture was a significant source of environmental contamination in Britain. Now that the factory has stopped using PFOA, further work is needed to test whether this association persists, and/or whether associations with replacement compounds (e.g., C6, or ether-PFAS) are now evident. The positive associations between most PFASs and the WWTW load, and arable land, suggest that the wastewater effluent and the spreading of sewage sludge, particularly in areas with low annual rainfall, are potentially important sources of PFAS contamination to freshwaters in England and Wales. Further research is needed in Britain to evaluate the concentrations of PFASs being discharged in the WWTW effluent and retained in sewage sludge, and the efficacy of policy relating to permissible concentrations. Additionally, further research is needed on methods to break down the stable carbon–fluorine bond—without which the cyclic nature of PFAS-contaminated sludge going to landfill and leachate going back to WWTW will continue (Figure 1).

### Biotic Factors

PFNA was the only compound associated with a biotic factor; otter body length was negatively correlated with the PFNA concentration (averaged model: *z* = 2.879, *p* < 0.01). Visualization of model predictions (Figure S5) suggested that sex might be a confounding variable, with the smaller otters in the study being predominantly female, and some having higher concentrations of PFNA than the males. Due to collinearity, we were not able to include both sex and length in the same model. Instead, we checked for an association with sex by exchanging the length for sex, and re-running all models: sex was not a significant term in any averaged model. This supports a previous study in Eurasian otters, which also showed a lack of sex difference in PFAS concentrations^60^ and also supports an inference here that sex does not confound the reported association between PFNA and length. Although all otters in our study were categorized as adults, previous analysis of age suggests these otters likely range between ca. 2 and 8 years,^83^ and larger otters are likely to be older. A decrease in long-chain PFCAs with age has been reported in bottlenose dolphins (*Tursiops truncatus*), and it was suggested this could be due to elimination via gestation and lactation (in females), enhanced elimination by older dolphins, and/or a change of diet with age.^84^ Previous research suggested maternal transfer is not a significant pathway of PFAA elimination in Eurasian otters (although the study only included one mother and cub).^60^ With all otters in our study being adults, and therefore all females showing signs of current or previous reproduction, we are unable to compare concentrations between age classes or between nulliparous and parous otters. Why this association with length was unique to PFNA (and not seen for other PFASs) is unclear. It is important to recognize this association between lengths, and only one compound could be a type II error and not be a true association; further research is needed to examine biotic associations (such as with age and reproduction) across a range of PFASs.

Statistical models showed no association between otter body condition and PFAS concentrations. This result supports findings from other studies on mustelids and marine mammals.^58,61,67^ PFASs are lipophobic and therefore do not concentrate in lipid-rich tissues, consequently lipid mobilization as a result of starvation does not appear to cause elevated liver concentrations of PFASs. A study comparing concentrations of PFASs in “lean” and “fat” Arctic fox (*Vulpes lagopus*) also found that the majority of PFAS hepatic concentrations showed no association with body condition, but PFNA, PFDA, and PFHpS were exceptions to this and were higher in lean foxes.^85^ The study on foxes^85^ and other studies^86^ have found associations between body condition and concentration of PFASs in other tissues, such as the adipose tissue and blood. Therefore, the inclusion of body condition in future studies of PFASs is important, to further explore associations that may differ between species, tissue matrices tested, and between different PFASs.

Overall, our study shows the widespread pollution of British freshwaters with PFASs and clearly demonstrates the otter as an effective sentinel species for PFAS contamination. Results support the need for an essential-use-only principle for PFASs^87^ and the management of PFASs as a class to reduce the cost and increase the speed of the regulatory process.^7^

## Supporting Information Available

Geographical locations of otters selected for analysis from England and Wales, histograms showing range of values for biotic and spatial variables used in statistical modeling, histograms of individual contaminant concentrations, model-predicted contaminant concentrations with an average WWTW load (measured in population equivalent) of WWTWs in the 10 km radius around the location of death of each otter, model-predicted contaminant concentrations with the percentage of arable land in the 10 km radius around the location of death of each otter, model-predicted PFNA concentrations with the otter length, descriptive statistics for each of the 15 PFASs analyzed, and GLM results for each of the 12 PFASs modeled (PDF)
