# Supplementary material for: Anthropogenic Drivers of Variation in Concentrations of Perfluoroalkyl Substances in Otters (Lutra lutra) from England and Wales
Source: Environ Sci Technol. 2022 Jan 11;56(3):1675–87. doi: 10.1021/acs.est.1c05410 (PMC8812117; doi:10.1021/acs.est.1c05410)
Supplement: Supplementary file 1 — es1c05410_si_001.pdf [file es1c05410_si_001.pdf]

Anthropogenic drivers of variation in concentrations of perfluoroalkyl substances (PFAS) in otters (*Lutra lutra*) from England and Wales

Emily O'Rourke[a], Juliet Hynes[a1] Sara Losada[b], Jonathan L Barber[b], M Glória Pereira[c], Eleanor F Kean[a], Frank Hailer[a] and Elizabeth A Chadwick[a]*

[a] Cardiff University, School of Biosciences, Museum Avenue, Cardiff, CF10 3AX

[b] Centre for Environment, Fisheries and Aquaculture Science (Cefas), Lowestoft, Suffolk

[c] UK Centre for Ecology and Hydrology, Lancaster Environment Centre, Library Avenue, Bailrigg, Lancaster LA1 4AP

*Corresponding author: ChadwickEA@cardiff.ac.uk

Supporting Information

Figures:

Figure S1: Geographical locations of otters selected for analysis from England and Wales.

Figure S2: Histograms showing range of values for biotic and spatial variables used in statistical modelling.

Figure S3: Histograms of individual contaminant concentrations.

Figure S4: Model predicted contaminant concentrations with average WWTW Load (measured in population equivalent) of WWTWs in 10km radius around location of death of each otter.

Figure S5: Model predicted contaminant concentrations with percentage of arable land in 10 km radius around location of death of each otter.

Figure S6: Model predicted PFNA concentrations with otter length.

Tables:

Table S1: Descriptive statistics for each of the 15 PFAS analysed.

Table S2: GLM results for each of the 12 PFAS modelled.

[1] Change of affiliation for Juliet Hynes: Gloucestershire Wildlife Trust, Conservation Centre, Robinswood Hill Country Park, Reservoir Road, Gloucester, GL4 6SX

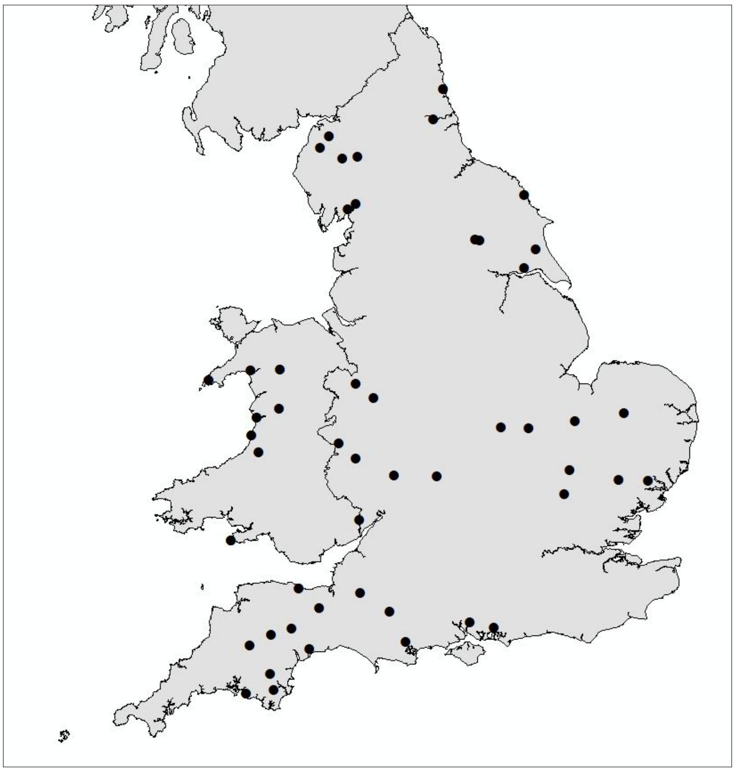

Figure S1: Geographical locations of otters selected for analysis from England and Wales. Mapped using ArcMap 10.5.1.

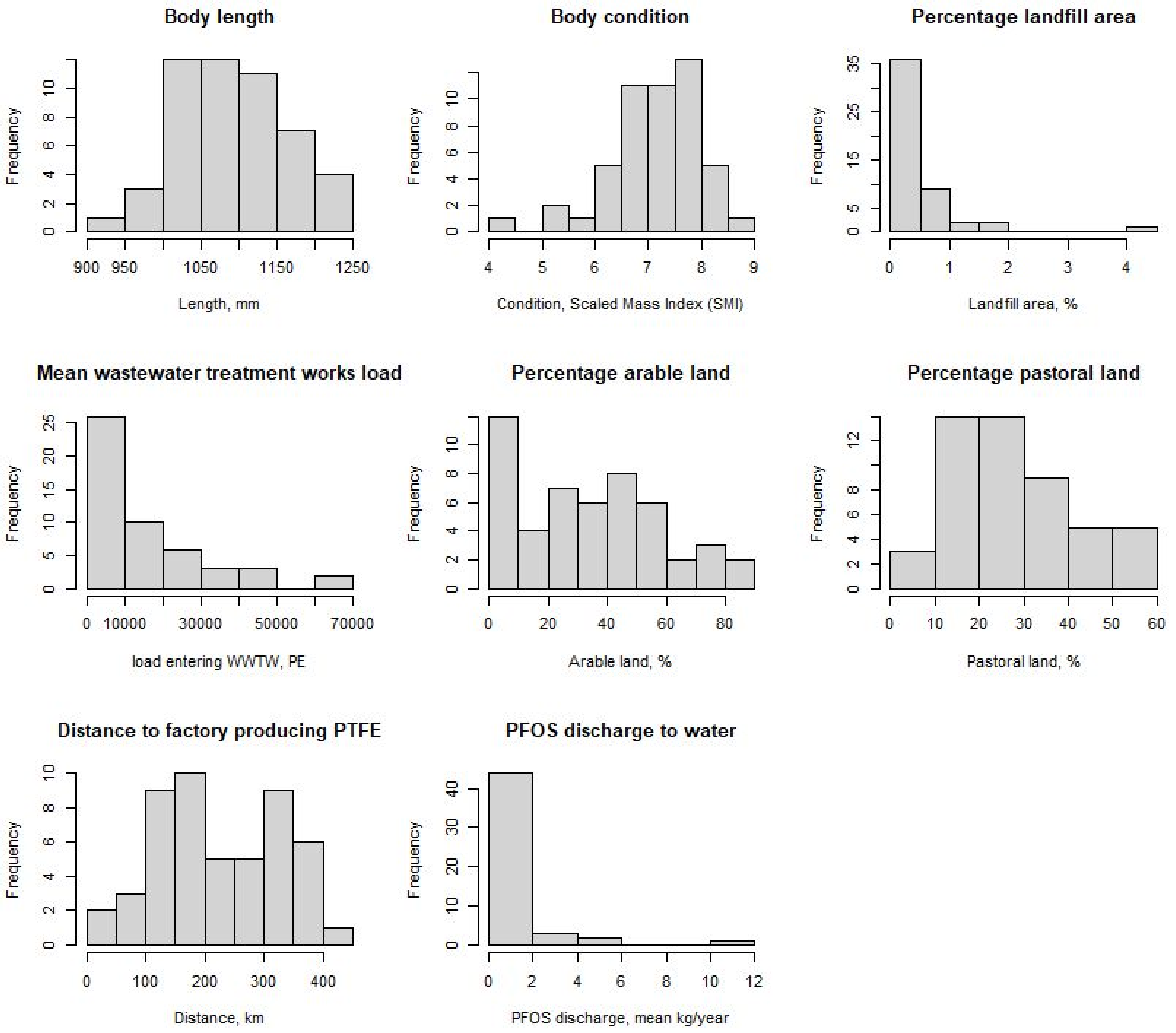

Figure S2: Histograms showing range of values for biotic and spatial variables used in statistical modelling.

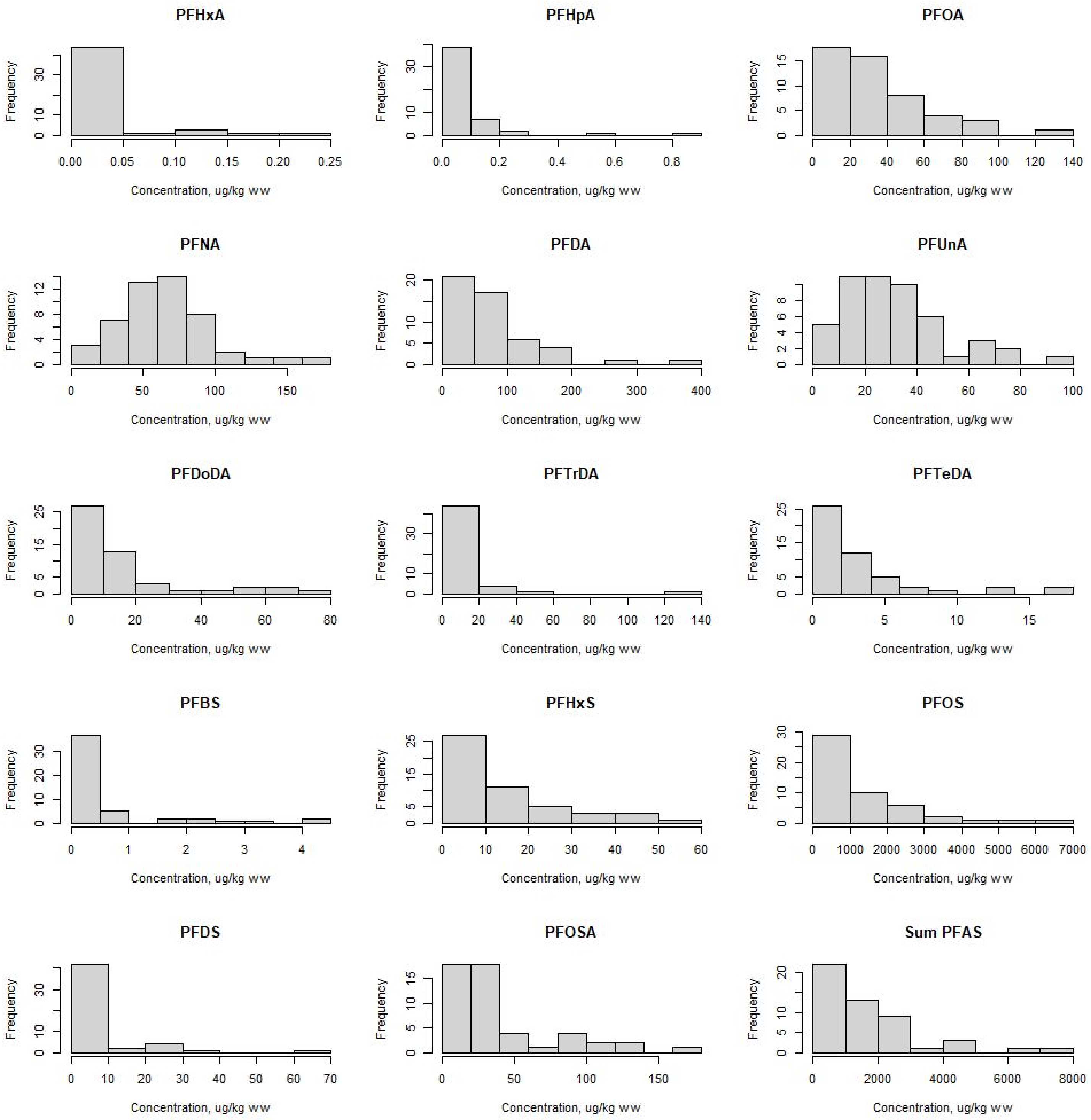

Figure S3: Histograms of individual contaminant concentrations. PFPeA not shown due to 0% detection.

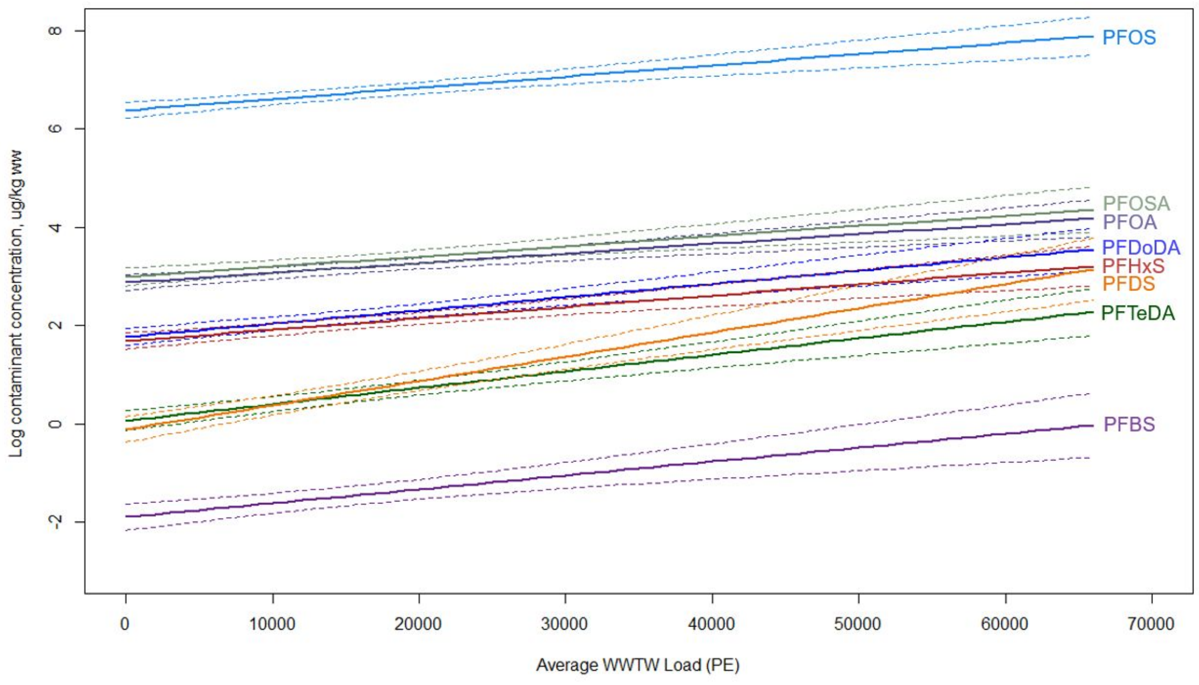

Figure S4: Model predicted contaminant concentrations (solid lines, ± SE) with average WWTW Load (measured in population equivalent) of WWTWs in 10km radius around location of death of each otter. Other variables in the model are controlled (see statistical methods).

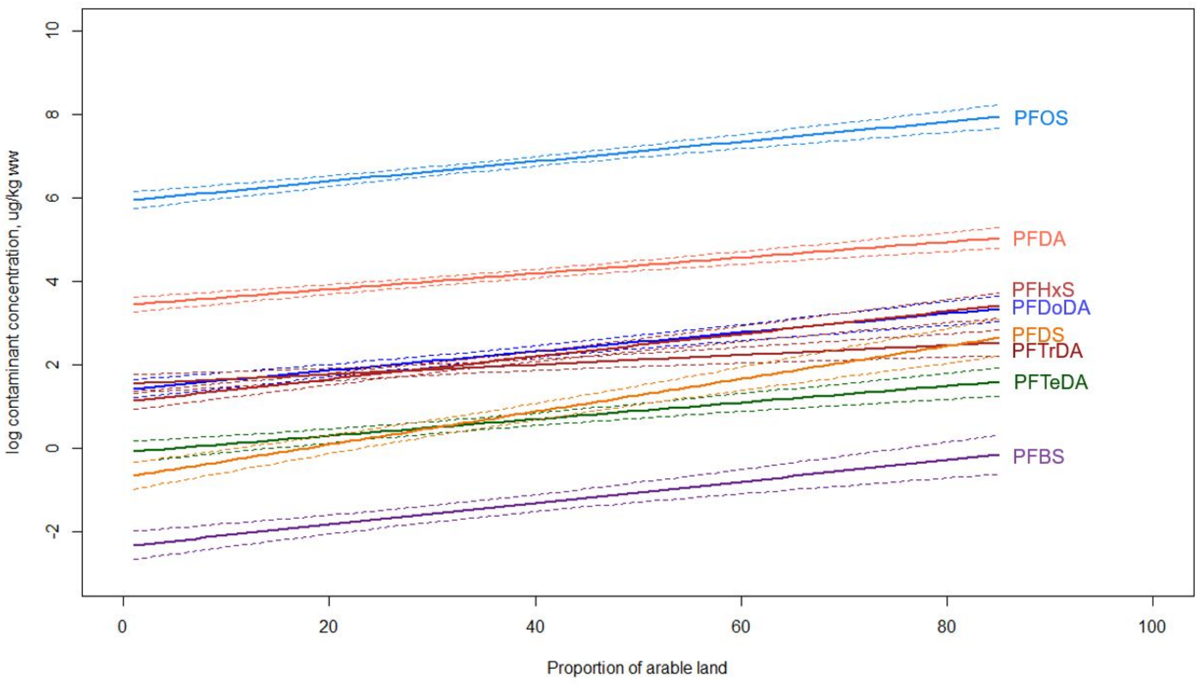

Figure S5: Model predicted contaminant concentrations (solid lines, ± SE) with percentage of arable land in 10 km radius around location of death of each otter. Other variables in the model are controlled (see statistical methods).

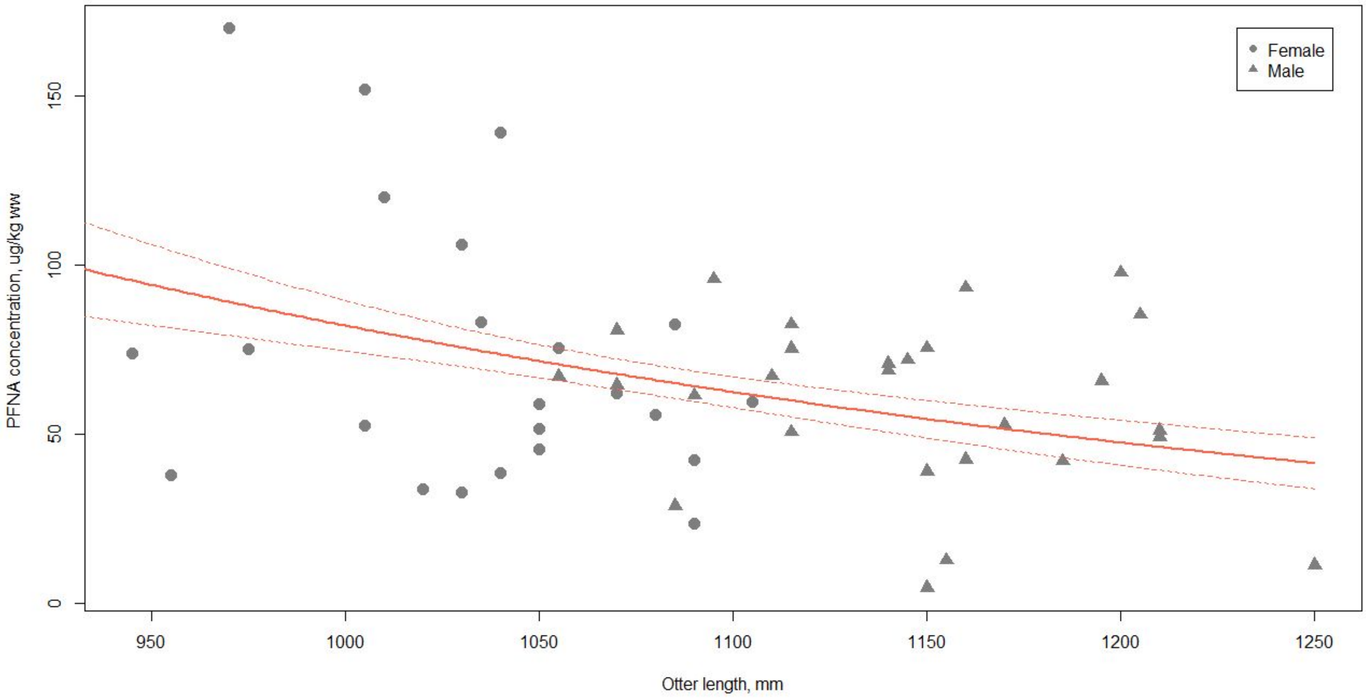

Figure S6: Model predicted PFNA concentrations (red lines, ± SE) with otter length. Females = circles, Males = triangles. Other variables in the model are controlled (arable land, pastoral land and landfill area, see statistical methods).

Table S1: Descriptive statistics for each of the 15 PFAS analysed. Concentrations are recorded in µg/kg ww, and reported to 3 significant figures. Compounds are grouped into their types; perfluoroalkyl carboxylic acids (PFCAs) and perfluoroalkyl sulfonic acids (PFSAs) and precursor compound perfluorooctane sulfonamide (PFOSA). Mean concentrations are provided for comparison with other studies, but are not considered a good measure of the average because of highly skewed data distributions.

Chemical abbreviation	Detection Frequency (%)	Minimum	Maximum	Median	Mean
		Perfluoroalkyl carboxylic acids			
PFPeA	0	<LOQ	<LOQ		
PFHxA	12	<LOQ	0.229	0.0250	0.0382
PFHpA	42	<LOQ	0.843	0.0250	0.0834
PFOA	100	1.76	130	27.2	34.1
PFNA	100	4.41	170	63.1	65.5
PFDA	100	6.55	369	70.9	81.4
PFUnA	100	4.5	96.2	25.4	31.1
PFDoDA	100	0.572	75.1	8.84	15.7
PFTrDA	100	1	140	6.70	11.9
PFTeDA	98	<LOQ	17.3	1.62	3.38
		Perfluoroalkyl sulfonic acids			
PFBS	80	<LOQ	4.44	0.287	0.657
PFHxS	100	1.03	55.2	7.09	13.3
PFOS	100	78.8	6800	820	1340
PFDS	98	<LOQ	63.8	1.87	6.96
		Precursor compound perfluorooctane sulfonamide			
PFOSA	100	3.35	171	28.8	41

Table S2: GLM results for each of the 12 PFAS modelled. Significance codes: '***' 0.001, '**' 0.01, '*' 0.05, '.' 0.1, ' ' 1. Standardised estimates presented for averaged models. Results of single top models for PFTeDA and PFDS presented. 'Estimate' is the estimated coefficient, and SE is the standard error around that estimate.

Dependent variable (number of top models)	Independent variables retained in top models	Estimate	SE	Adjusted SE	Z value	P value	Relative variable importance
PFOA	WWTW	0.6241	0.2251	0.2311	2.701	0.00692 **	1.00
(2 models)	PTFE	-0.7960	0.2342	0.2402	3.314	0.00092 ***	1.00
	Arable	0.1121	0.2036	0.2065	0.543	0.58712	0.39
PFNA	Length	-0.39733	0.13450	0.13799	2.879	0.00398 **	1.00
(4 models)	Pastoral	-0.21849	0.17548	0.17804	1.227	0.21976	0.78
	Arable	0.14322	0.15661	0.15859	0.903	0.36646	0.63
	Log Landfill	-0.02811	0.08534	0.08633	0.326	0.74476	0.16
PFDA	Arable	0.89714	0.20662	0.21201	4.232	2.32e-05 ***	1.00
(3 models)	WWTW	0.32945	0.24268	0.24632	1.337	0.181	0.81
	Length	-0.09509	0.17804	0.18027	0.527	0.598	0.35
PFUnA	Arable	0.39140	0.23062	0.23455	1.669	0.0952 .	0.90
(8 models)	Condition	0.15877	0.21449	0.21691	0.732	0.4642	0.50
	Length	-0.07109	0.15553	0.15758	0.451	0.6519	0.31
	WWTW	0.07001	0.14805	0.15010	0.466	0.6409	0.30
PFDoDA	Arable	1.0788	0.2634	0.2704	3.990	6.59e-05 ***	1.00

(2 models)	WWTW	0.8518	0.2597	0.2666	3.194	0.0014 **	1.00
	Length	-0.1048	0.2119	0.2151	0.487	0.6259	0.36
PFTrDA	Arable	0.5495	0.2593	0.2661	2.065	0.0389 *	1.00
(2 models)	WWTW	0.2843	0.3018	0.3053	0.931	0.3518	0.62
PFBS	Arable	1.2194	0.3946	0.4050	3.011	0.00261 **	1.00
(2 models)	WWTW	0.8934	0.3907	0.4011	2.227	0.02593 *	1.00
	Length	-0.0981	0.2652	0.2701	0.363	0.71644	0.30
PFHxS	Arable	1.28235	0.26060	0.26696	4.804	1.6e-06 ***	1.00
(7 models)	WWTW	0.73172	0.24356	0.24997	2.927	0.00342 **	1.00
	Pastoral	-0.11045	0.20990	0.21276	0.519	0.60366	0.35
	Condition	-0.07694	0.17948	0.18181	0.423	0.67217	0.26
	Length	0.06278	0.16282	0.16507	0.380	0.70368	0.23
	Log Landfill	-0.02865	0.13026	0.13236	0.216	0.82864	0.11
PFOS	Arable	1.12580	0.23639	0.24245	4.644	3.4e-06 ***	1.00
(3 models)	WWTW	0.72281	0.23639	0.24247	2.981	0.00287 **	1.00
	Length	0.04191	0.13349	0.13590	0.308	0.75775	0.23
	Log Landfill	-0.05094	0.16669	0.16974	0.300	0.76410	0.22
PFOSA	WWTW	0.65739	0.27871	0.28541	2.303	0.0213 *	1.00
(6 models)	Condition	-0.17593	0.25841	0.26159	0.673	0.5012	0.47
	Log Landfill	-0.12790	0.24507	0.24813	0.515	0.6063	0.34
	Arable	-0.02113	0.10307	0.10479	0.202	0.8402	0.10
	Length	-0.02299	0.10915	0.11091	0.207	0.8358	0.10

Dependent variable	Independent variables	Estimate	SE	T value	P value
PFTeDA	WWTW	3.352e-05	9.159e-06	3.660	0.000637 ***
(Single top model)	Arable	1.988e-02	6.105e-03	3.256	0.002099 **
PFDS	WWTW	4.934e-05	1.200e-05	4.111	0.000157 ***
(Single top model)	Arable	3.927e-02	7.999e-03	4.909	1.15e-05 ***
